# SPC25 overexpression promotes tumor proliferation and is prognostic of poor survival in hepatocellular carcinoma

**DOI:** 10.18632/aging.202329

**Published:** 2020-12-19

**Authors:** Baozhu Zhang, Qing Zhou, Qiankun Xie, Xiaohui Lin, Wenqiang Miao, Zhaoguang Wei, Tingting Zheng, Zuoliang Pang, Haosheng Liu, Xi Chen

**Affiliations:** 1 Department of Oncology, People’s Hospital of Shenzhen Baoan District, The Affiliated Baoan Hospital of Shenzhen, Southern Medical University, Shenzhen 518101, Guangdong, China; 2 Department of Core Facility, People’s Hospital of Shenzhen Baoan District, The Affiliated Baoan Hospital of Shenzhen, Southern Medical University, Shenzhen 518101, Guangdong, China; 3 Department of Radiation Oncology, Nanfang Hospital, Southern Medical University, Guangzhou 510515, Guangdong, China

**Keywords:** SPC25, HCC prognosis, HCC progression

## Abstract

Background: The nuclear division cycle 80 (NDC80) complex assures proper chromosome segregation during the cell cycle progression. SPC25 is a crucial component of NDC80, and its role in hepatocellular carcinoma (HCC) has been explored recently. This study characterized the differential expression of SPC25 in HCC patients of different races and HBV infection status.

Methods: Expression patterns of SPC25 were evaluated in TCGA and Chinese HCC patients. Kaplan-Meier analysis was applied to examine the predictive value of SPC25. *In vitro* and *in vivo* functional assays were conducted to explore the role of SPC25 in HCC. Bioinformatics methods were applied to investigate the regulatory mechanisms of SPC25.

Findings: The mRNA levels of SPC25 were up-regulated in HCC. SPC25 has a significantly higher transcriptional level in Asian patients than Caucasian patients. SPC25 promoted HCC cell proliferation *in vitro* and tumor growth *in vivo* by accelerating the cell cycle. We identified transcription factors, miRNAs, and immune cells that may interact with SPC25.

Interpretation: The findings suggest that increased expression of SPC25 is associated with poor prognosis of HCC and enhances the proliferative capacity of HCC cells. SPC25 could serve as a valuable prognostic marker and a novel treatment target for HCC.

## INTRODUCTION

In 2017, 953, 000 new liver cancer cases and 819, 000 liver cancer deaths occurred globally [1]. Of note, hepatocellular carcinoma (HCC) cases from less developed regions of the world, including East Asia and North Africa, accounted for nearly four out of five (83%) of the total cases [2, 3]. HCC is associated with a poor prognosis due to deficiency in targeted screening, lack of early diagnosis, and few effective treatment options. Therefore, it is critical to identify novel diagnostic biomarkers and therapeutic targets to improve HCC screening, diagnosis, and treatment.

The nuclear division cycle 80 (NDC80) complex is a conserved heterotetramer that ensures accurate microtubule-kinetochore attachment [4]. The NDC80 complex consists of NDC80, Nuf2 (CDCA1), spindle component 24 (SPC24), and spindle component 25 (SPC25) [5]. Deregulation of the components of this complex can lead to uncontrolled proliferation and reduced apoptosis [6]. Overexpression of NDC80 was associated with poor prognosis in various tumors, including gastric, colorectal, and pancreatic cancer [7–10]. Silencing of Nuf2 suppressed tumor growth in glioma, pancreatic cancer, and colon cancer cells [11–13]. In breast, lung, and anaplastic thyroid cancer, SPC24 plays a role in promoting cancer progression [14–16]. Moreover, the up-regulation of SPC25 was observed in lung cancer, prostate cancer, and breast cancer, significantly enhancing the proliferation of these tumor cells [17–21]. Elevated expression of the NDC80 complex components, NDC80/Nuf2/SPC24/SPC25, predicts poor survival in HCC [22]. Recent literature reported that overexpression of SPC25 was related to poor prognosis of HCC via promoting tumor growth and metastasis [23, 24]. However, the detailed expression status of SPC25 in HCCC and the biological function of SPC25 in HCC cells remain unclear.

In our previous study, we examined the mRNA level, clinical value, and function of SPC25 in HCC. Results showed that SPC25 is frequently up-regulated in HCC. Functional studies demonstrate that SPC25 enhances both *in vitro* and *in vivo* tumor growth of HCC by accelerating the cell cycle.

## RESULTS

### SPC25 expression was up-regulated in HCC samples

**Figure 1:**
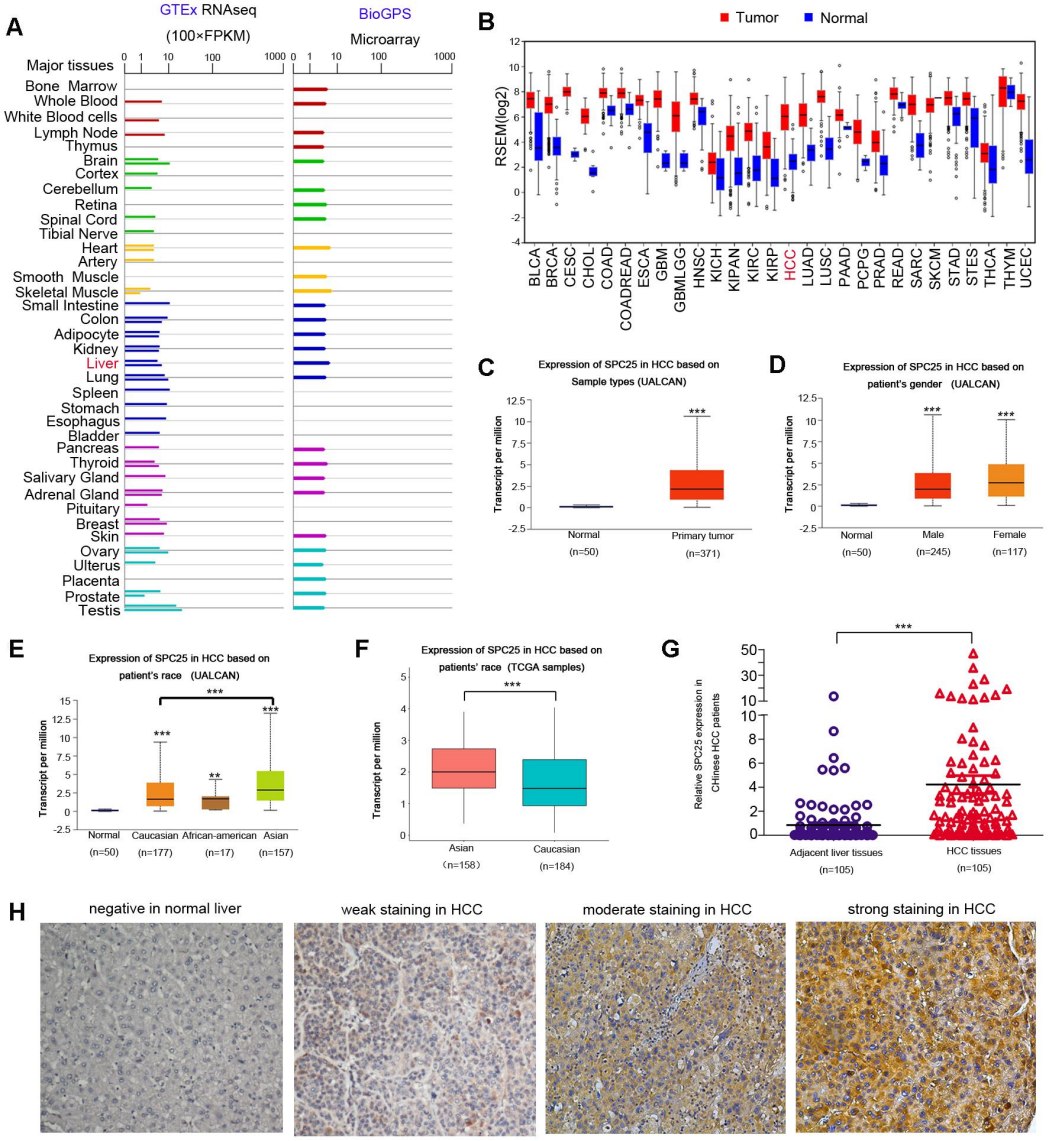
**SPC25 expression in normal human tissue, human tumors, and in HCC.** (**A**) SPC25 mRNA expression in normal human tissues. GTEx: The Genotype-Tissue Expression project; FPKM: Fragments Per Kilobase per Million. (**B**) SPC25 mRNA expression in multiple human cancers is higher compared to corresponding normal tissues. RPKM: Reads Per Kilobase per Million. (**C**) SPC25 mRNA is significantly overexpressed in HCC samples compared to normal controls, according to UALCAN. (**D**) Both male and female HCC patients showed a higher SPC25 transcriptional level than normal controls, according to UALCAN. (**E**) UALCAN analysis showed that SPC25 mRNA levels are significantly higher among Caucasian, African-American, and Asian HCC patients than normal controls. Besides, SPC25 showed a relatively higher transcriptional level in Asian patients in contrast with Caucasian patients. (**F**) SPC25 mRNA expression in Asians HCC patients is significantly higher than that in the Caucasian HCC patients based on TCGA HCC samples screened by race. (**G**) SPC25 mRNA is significantly overexpressed in Chinese HCC tumor specimens vs. corresponding adjacent normal liver tissue. (**H**) Representative images of SPC25 protein expression in normal liver and HCC samples from Chinese patients. (*P < 0.05, **P < 0.01, ***P < 0.001).

Data from the Genotype-Tissue Expression (GTEx) project and BioGPS revealed that *SPC25* mRNA existed in most normal human tissues (Figure 1A). According to UALCAN, SPC25 mRNA expression was notably higher in most human cancer than in the corresponding normal tissue (Figure 1B). HCC has several notable epidemiologic features, including marked variations between men and women, geographic regions, racial and ethnic groups [25]. Therefore, more comprehensive expression analysis of SPC25 mRNA in HCC was characterized using UALCAN. Compared with healthy controls, SPC25 mRNA levels are significantly higher in HCC patients (Figure 1C). Either male or female patients showed a higher SPC25 level than normal controls (Figure 1D). In particular, SPC25 expression was significantly higher in Asian patients than Caucasian patients (Figure 1E). This result was further validated using a TCGA cohort containing 158 Asian patients and 184 Caucasian patients (Figure 1F).

To further characterize SPC25 mRNA expression status, qRT-PCR was performed using 105 pairs of HCC specimens from Chinese patients. Elevated SPC25 mRNA levels (defined as a two-fold increase) were detected in 72/105 (68.6%) of HCC tissues compared to matched non-tumor tissue. The average mRNA level of SPC25 in HCC was significantly higher when compared with that in the non-tumor tissue (4.12 vs. 1, respectively, P < 0.0001, paired student's t-test; Figure 1G).

We performed immunohistochemical (IHC) staining with 223 paired Chinese HCC tissue specimens and their adjacent normal liver tissues. SPC25 was barely detectable in normal liver tissue (Figure 1H). In contrast, SPC25 protein expression in Chinese HCC tissues exhibited varying staining intensity, from weak staining to intense staining (Figure 1H). Increased SPC25 protein levels were detected in 151/223 (67.7%) of informative LIHC tissues compared with adjacent non-tumor tissue.

### Prognostic value of SPC25 mRNA levels in HCC

**Figure 2:**
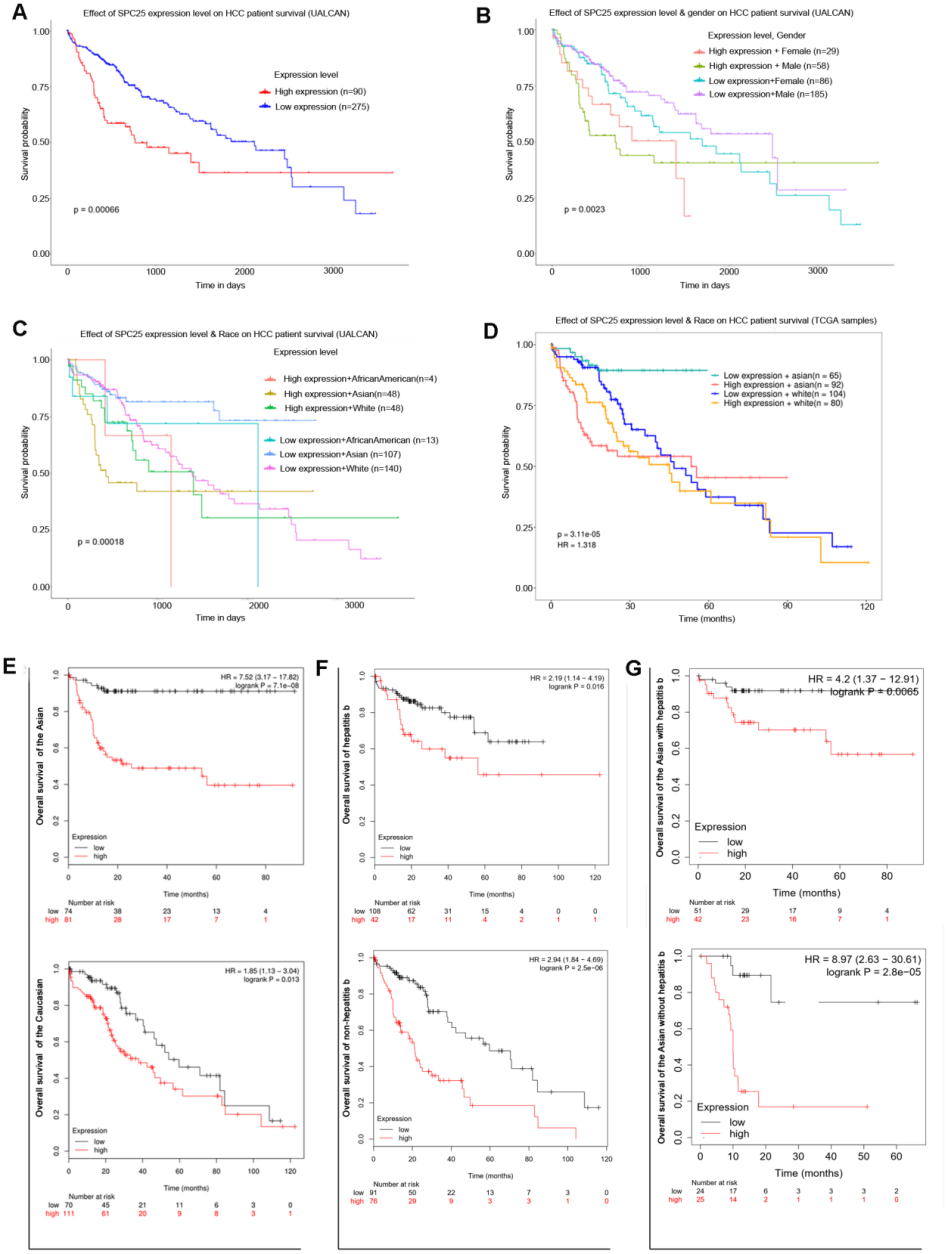
**Prognostic role of SPC25 mRNA in HCC patients.** (**A**) Effect of SPC25 expression level on HCC patient survival (UALCAN). (**B**) Effect of SPC25 expression level and gender on HCC patient survival (UALCAN). (**C**) Effect of SPC25 expression level and Race on HCC patient survival (UALCAN). (**D**) Effect of SPC25 expression level and Race on HCC patient survival (TCGA samples). (**E**) Subgroup overall survival analysis of SPC25 mRNA level in Asian or Caucasian HCC patients. (**F**) Subgroup overall survival analysis of SPC25 mRNA level in HCC patients with or without hepatitis b. (**G**) Subgroup overall survival analysis of SPC25 mRNA level in Asian HCC patients with or without hepatitis b.

Online analysis of UALCAN showed that patients with high *SPC25* transcriptional levels had shorter overall survival (OS) than low level (Figure 2A). In the Expression level and Gender subgroup, male patients with low SPC25 level have the most prolonged OS, while male patients with high levels have the shortest (Figure 2B). In the Expression level and Race subgroup, Asian patients with low SPC25 levels have a survival advantage over Caucasian patients with low levels, Caucasian patients with high levels, and Asian patients with high levels (Figure 2C). Survival analysis of TCGA samples further demonstrated that high SPC25 expression caused a more significant survival disadvantage in Asian patients than Caucasian patients (Figure 2D).

Kaplan-Meier Plotter was used further to explore the survival differences between two specific subgroups. Results displayed that Asian patients with high SPC25 level have poorer OS than low level and the hazard ratio (HR) was 7.52 (95%CI: 3.17-17.82, P < 0.001, Figure 2E); High SPC25 level brought a survival disadvantage in Caucasian patients with a much lower HR-1.85 (95%CI: 1.13-3.04, P = 0.013, Figure 2E). As to hepatitis virus B (HBV) infection status, subgroup analysis exhibited that OS of high SPC25 level in patients with HBV infection is shorter than low level (HR:2.19, 95%CI: 1.14-4.19, P = 0.016, Figure 2F). In HCC patients with no HBV infection, high SPC25 level caused a slightly higher OS disadvantage (HR:2.94, 95%CI:1.84-4.69, P < 0.001, Figure 2F). In Asia, chronic HBV infection is the dominant risk factor for HCC [25]. Accordingly, the relationship of SPC25 expression pattern and OS in Asian patients with or without HBV infection was checked. Result showed that high SPC25 expression caused a higher HR in Asian patients without HBV infection (HR:8.97, 95%CI:2.63-30.61, P < 0.001, Figure 2F) than those patients with HBV infection (HR:4.2, 95%CI:1.37-12.91, P = 0.007, Figure 2G).

### Correlations between SPC25 mRNA expression and clinicopathological features

Demographic and clinicopathological parameters of 342 HCC patients are given in Table 1. The high SPC25 expression was significantly correlated with male (P = 0 .039, Table 1), high pathologic stage (P = 0 .002, Table 1), high T stage (P = 0 .003, Table 1), Asian race (P = 0 .007, Table 1), high histologic grade (P < 0.001, Table 1).

**Table 1:** Association of SPC25 mRNA expression with clinicopathological features in HCC (TCGA samples).

**Clinical features**	**Cases**		**SPC25 expression**	***P* value**
	**low level (171)**	**high level (171)**		
***Age (years old)***				0.148
<60	157	86(54.7%)	71(45.2%)	
≥60	184	84(45.7%)	100(54.3%)	
***Gender***				0.039
Male	115	67(58.3%)	48 (41.7%)	
Female	227	104(34.4%)	123(65.6%)	
***Pathologic stage***				0.002
Stage I	157	62(39.5%)	95(60.5%)	
Stage II	80	45(56.3%)	35(43.7%)	
Stage III	81	53(65.4%)	28(34.6%)	
Stage IV	6	2(33.3%)	4(66.7%)	
***T stage***				0.003
T0	1	0(0%)	1(100%)	
T1	165	65(39.4%)	100(60.6%)	
T2	86	49(57.0%)	37(43.0%)	
T3	76	49(64.5%)	27(35.5%)	
T4	13	8(61.5%)	5(38.5%)	
***N stage***				0.705
N0	239	123(51.5%)	116(48.5%)	
N1	4	2(50.0%)	2(50.0%)	
NX	87	40(46.0%)	47(54.0%)	
***M stage***				0.389
M0	152	59(38.8%)	93(61.2%)	
M1	71	13(18.3%)	58(81.7%)	
***Race***				
Asian	88	22(25.0%)	66(75.0%)	0.007
White	135	40(29.6%)	95(70.4%)	
***Histologic Grade***				
G1	48	13(27.0%)	35(73.0%)	< 0.001
G2	163	70 (42.9%)	93(57.1%)	
G3	115	76 (66.0%)	39 (33.9%)	
G4	12	9 (75.0%)	3 (25.0%)	

Statistical significance (*P* < 0.05) is shown in bold.

### High SPC25 expression were independent predictive factors for poor outcomes in HCC patients

**Figure 3:**
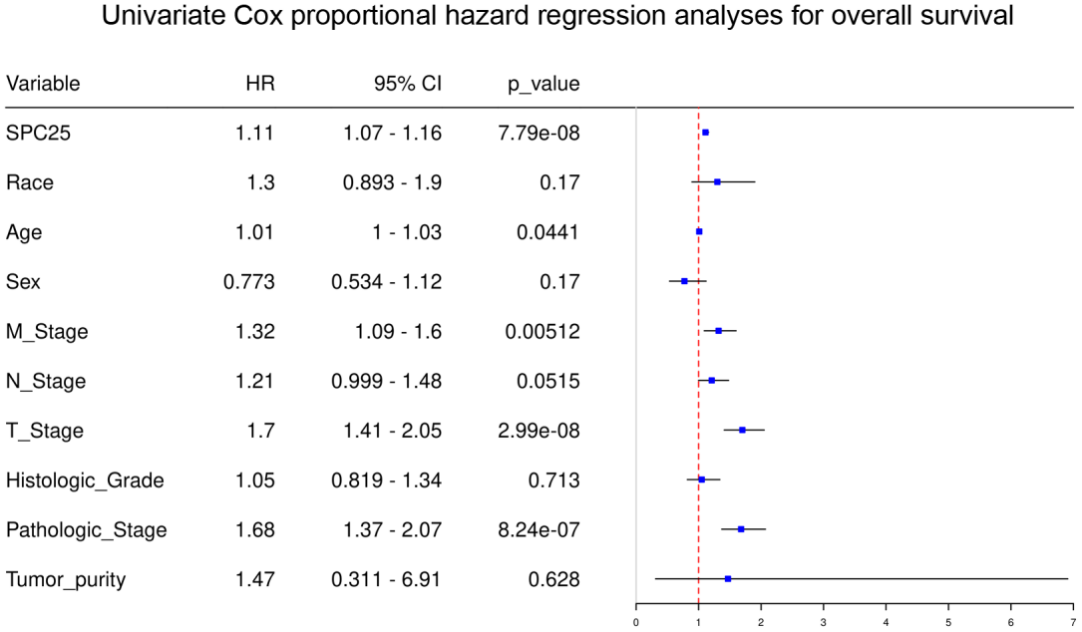
**Univariate regression analysis of clinical information in TCGA HCC samples.**

Univariate analysis demonstrated that high SPC25 mRNA level (P < 0.001), Age (P=0.044), M-stage (P < 0.001), T-stage (P < 0.001), and Pathologic stage (P < 0.001) were independent prognostic factors for HCC patients (Figure 3). In contrast, Race, Sex, N-stage, Histologic-Grade, and Tumor-purity were not independent prognostic factors for HCC patients (Figure 3).

### Ectopic overexpression of SPC25 enhances HCC tumor growth

**Figure 4:**
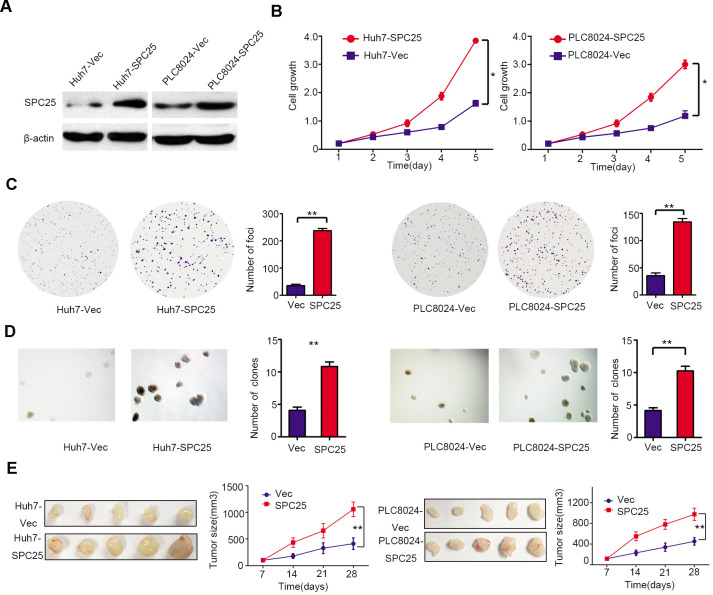
**Functional analysis of SPC25 overexpression in HCC cells.** (**A**) The protein levels of SPC25 were detected by western blot analysis in SPC25 or vector-transfected cells. β-actin was used as an endogenous control. (**B**) CCK-8 assays, (**C**) colony formation, and (**D**) non-adherent colony formation assays demonstrated that overexpression of SPC25 promoted proliferation of Huh7 and PLC8024. (**E**) Images of xenograft tumors derived from SPC25-transfected cells and their vectors in nude mice. Tumor sizes were compared in the right chart. (*P < 0.05, **P < 0.01, ***P < 0.001).

To characterize the role of SPC25 in HCC tumorigenicity, *SPC25* was stably transfected into Huh7 and PLC8024 (Huh7-SPC25 and PLC8024-SPC25) cells. Cells transfected with empty vector (Huh7-Vec and PLC8024-Vec) were used as controls. Western blot analysis was used to confirm the efficiency of SPC25 transfection (Figure 4A). Cell growth, foci formation, and non-adherent colony formation assays were carried out to evaluate the *in vitro* function of SPC25. SPC25-transfected cells showed faster cell growth rate (Figure 4B), higher foci formation frequency (Figure 4C), and more vital colony formation ability in soft agar (Figure 4D) than control cells. To further study the effect of *SPC25* *in vivo*, the left and right dorsal flanks of nude mice (n = 5) were injected subcutaneously with empty vector and SPC25-transfected cells, respectively. Xenograft tumors developed from SPC25-transfected cells grew larger than tumors derived from empty vector-transfected cells (Figure 4E). Both *in vitro* and *in vivo* assays showed that overexpression of *SPC25* significantly promotes HCC tumor growth.

### DNA methylation and transcription factors that may affect SPC25 mRNA levels

**Figure 5:**
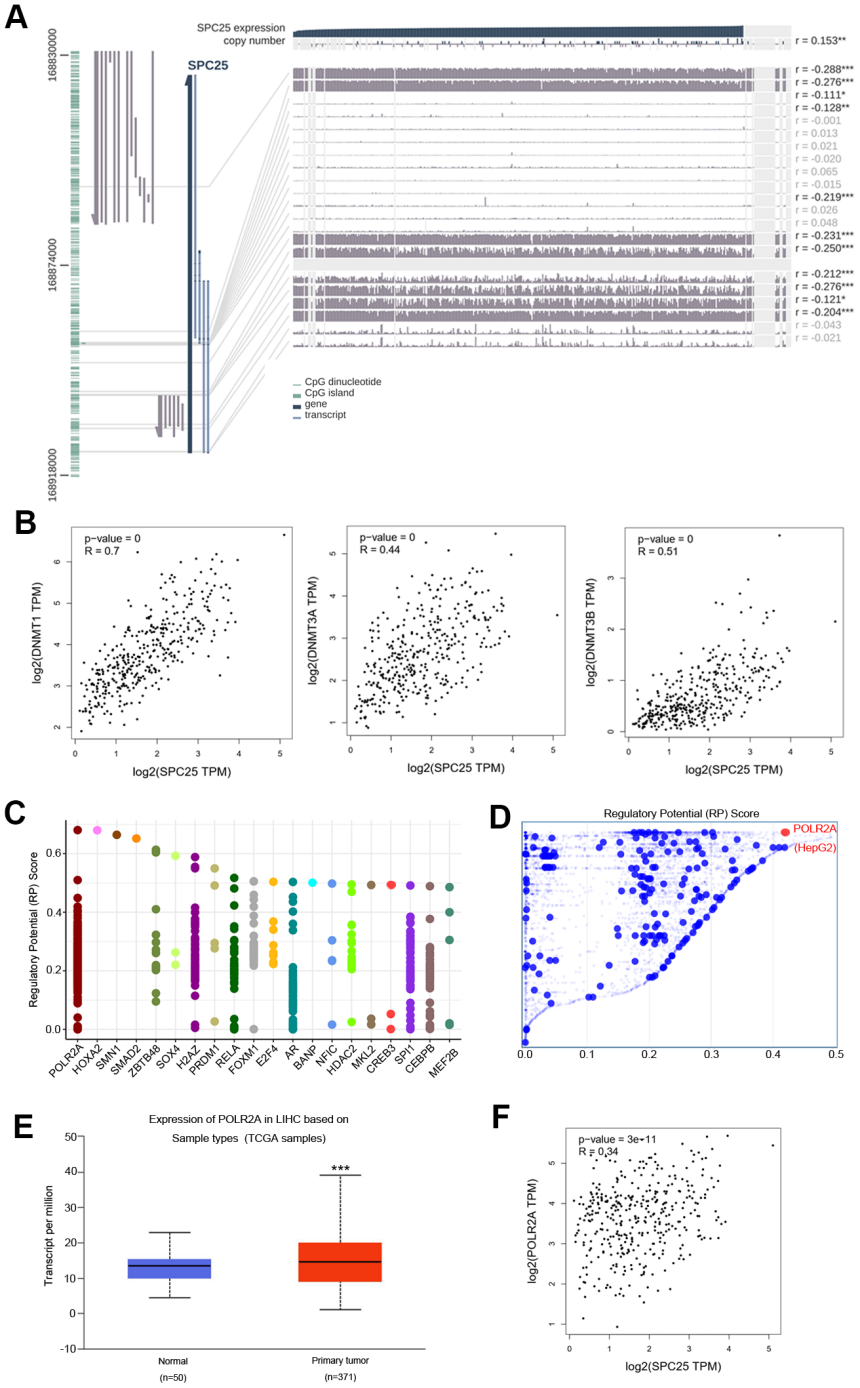
**DNA methylation modification and transcription factors associated with SPC25 in HCC.** (**A**) SPC25 DNA methylation modification in HCC. (**B**) Correlation between SPC25 mRNA expression and DNA methyltransferase (DNMT) expression. (**C**) Top 20 transcription factors (TFs) that potentially regulate SPC25 in HCC. (**D**) TFs with high regulatory potential in HepG2 cell lines (10k distance to transcription start site, TSS). (**E**) POLR2A mRNA expression is significantly higher in HCC samples than in normal samples. (**F**) Correlation between SPC25 and POLR2A mRNA expression. (*P < 0.05, **P < 0.01, ***P < 0.001).

DNA methylation modifications are extensively involved in regulating physiological and pathological pathways in HCC [26, 27]. We explored the DNA methylation status of the SPC25 gene in HCC using MEXPRESS. Several methylated sites were detected in the promoter region of the SPC25 gene (Figure 5A). Furthermore, we found a positive relationship between SPC25 mRNA expression and DNA methyltransferase (DNMT) mRNA expression levels in HCC (Figure 5B).

The top 20 regulatory TFs in human cancers were acquired using the Cistrome DB Toolkit (Figure 5C). We then evaluated these TFs in the HepG2 cell line. POLR2A ranked as the most likely potential regulatory factor in the HEPG2 cell line (Figure 5D). Gene expression of POLR2A in HCC was evaluated using UALCAN. POLR2A was significantly overexpressed in HCC (P < 0.0001, Figure 5E). Further correlation analysis using the GEPIA database indicated that POLR2A expression is significantly associated with SPC25 expression (P < 0.0001, r = 0.34, Figure 5F). These results indicate that SPC25 expression may increase when POLR2A is overexpressed.

### miR-451a negatively regulates SPC25 mRNA levels

**Figure 6:**
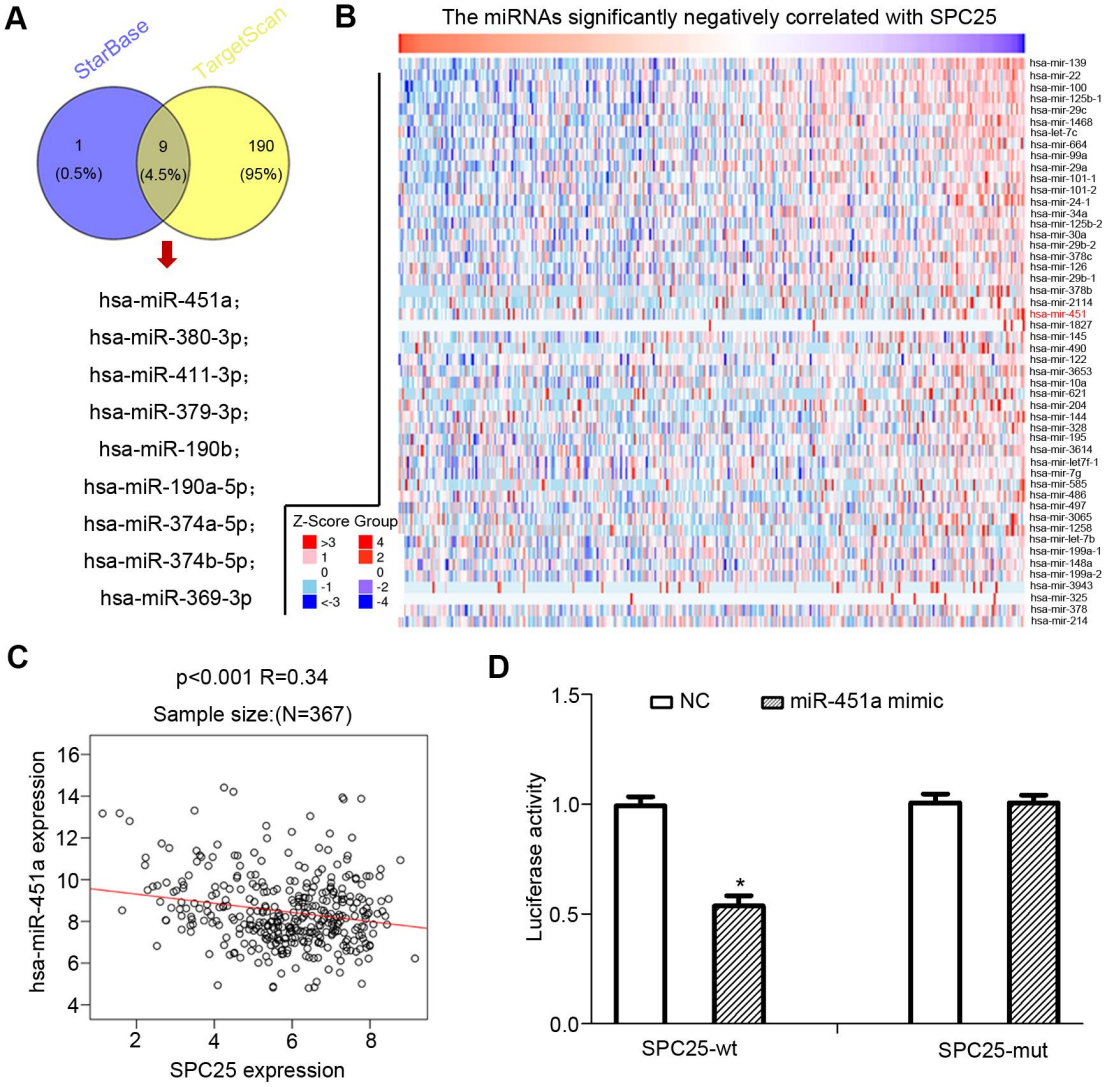
**miRNAs that down-regulated SPC25 expression in HCC.** (**A**) Two miRNA prediction datasets, starBase, and Targetscan were used to predict miRNAs that may bind to SPC25 mRNA. (**B**) miRNAs negatively correlated with SPC25 mRNA expression (data from Linkomics). (**C**) Correlation between SPC25 mRNA and miR-451 expression. (**D**) Dual-luciferase reporter assay for confirmation of the targeting relationship between miR-451a and SPC25; *P < 0.05; statistical data were presented as mean ± standard deviation; data between two groups were analyzed by paired t-test; one-way analysis of variance was used for multi-group comparisons; the experiment was repeated three times independently, NC negative control.

By searching starBase and Targetscan, we found nine miRNAs that were commonly predicted to bind to and down-regulate SPC25 expression in HCC: hsa-miR-369-3p, hsa-miR-369-3p, hsa-miR-374a-5p, hsa-miR-380-3p, hsa-miR-451a, hsa-miR-379-3p, hsa-miR-411-3p, hsa-miR-190b, hsa-miR-374b-5p, hsa-miR-1276 (Figure 6A). LinkedOmics was used to identify miRNAs negatively correlated with SPC25 expression in HCC. Only one of the nine SPC25-regulating miRNAs, hsa-miR-451a, was identified in the expression correlation heatmap (Figure 6B). Correlation analysis by LinkedOmics found a negative relationship between hsa-miR-451a expression and *SPC25* mRNA levels (Figure 6C).

Based on the dual-luciferase reporter gene assay analysis, luciferase activity decreased in the miR-137-Wt group co-transfected with SPC25-wt and miR-451a mimic, relative to the NC group (p < 0.05; Figure 6D). We found no notable difference in the luciferase activity in cells co-transfected with SPC25-Mut and miR-451a mimic (p > 0.05; Figure 6D).

### Mechanistic investigation into how SPC25 promotes HCC proliferation

**Figure 7:**
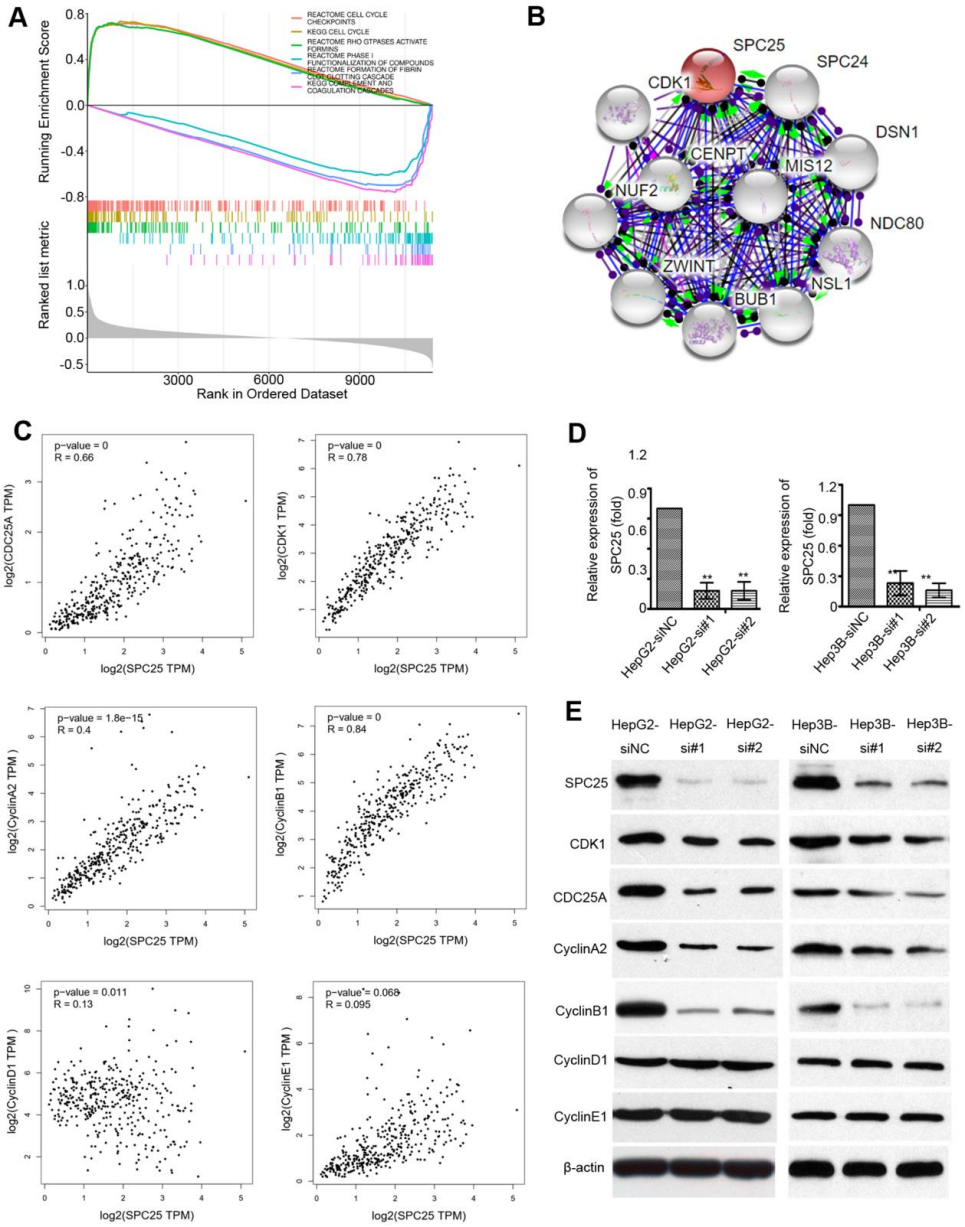
**Mechanistic investigation into how SPC25 promotes HCCs growth.** (**A**) Gene Set Enrichment Analysis (GSEA) shows statistically significant different biological function between subgroups of high SPC25 level and low SPC25 level. (**B**) STRING analysis of interactions between SPC25 and other proteins. (**C**) Correlation between SPC25 and cell cycle-regulation related genes, including CDK1, cdc25A, Cyclin A2, Cyclin B1, Cyclin D1, Cyclin E1. (**D**) Two siRNAs (si#1 and si#2) against SPC25 effectively silenced SPC25 expression, as determined by qRT-PCR. Negative control siRNA (siNC) and β-actin were used as negative and endogenous controls, respectively. The data are represented as the mean ± s.d. of three independent experiments. **P < 0.01. (**E**) Silencing SPC25 decreased the protein level of SPC25, CDK1, cdc25A, Cyclin A2, Cyclin B1, Cyclin D1, and Cyclin E1.

The MSigDB database was used for gene set enrichment analysis (GSEA). The TCGA samples were divided into two groups of high SPC25 expression and low SPC25 expression to study the significance of various functional sets of these two different groups. The result showed that high SPC25 expression was significantly associated with the cell cycle checkpoints (Figure 7A). Proteins that could bind to SPC25 were also characterized by a STRING interactive network (Figure 7B). Correlation analysis by GEPIA demonstrated that the transcriptional level of CDK1, cdc25A, CyclinA2, and CyclinB1 are significantly associated with SPC25 while CyclinD1 and CyclinE1 are not (Figure 7C).

To verify the role of SPC25 in cell growth, we knocked down SPC25 by siRNA in HCC cell lines HepG2 and Hep3B. Figure 7D showed that more than 80% of the SPC25 mRNA was silenced in HepG2 and Hep3B cells by si#1 and si#2. To further determine the effect of SPC25 on the cell cycle, the expression of cell cycle-associated genes, including CDK1, cdc25A, CyclinA2, CyclinB1, CyclinD1, and CyclinE1, were analyzed by western blot. Expression of CDK1, cdc25A, CyclinA1, and CyclinB2 significantly decreased after SPC25 knockdown with siR#1 and siR#2, while CyclinD1 and CyclinE1 showed no noticeable change (Figure 7E). These data demonstrate that silencing SPC25 inhibited the cell-cycle in HCC cells.

### SPC25 expression is related to immune cells

**Figure 8:**
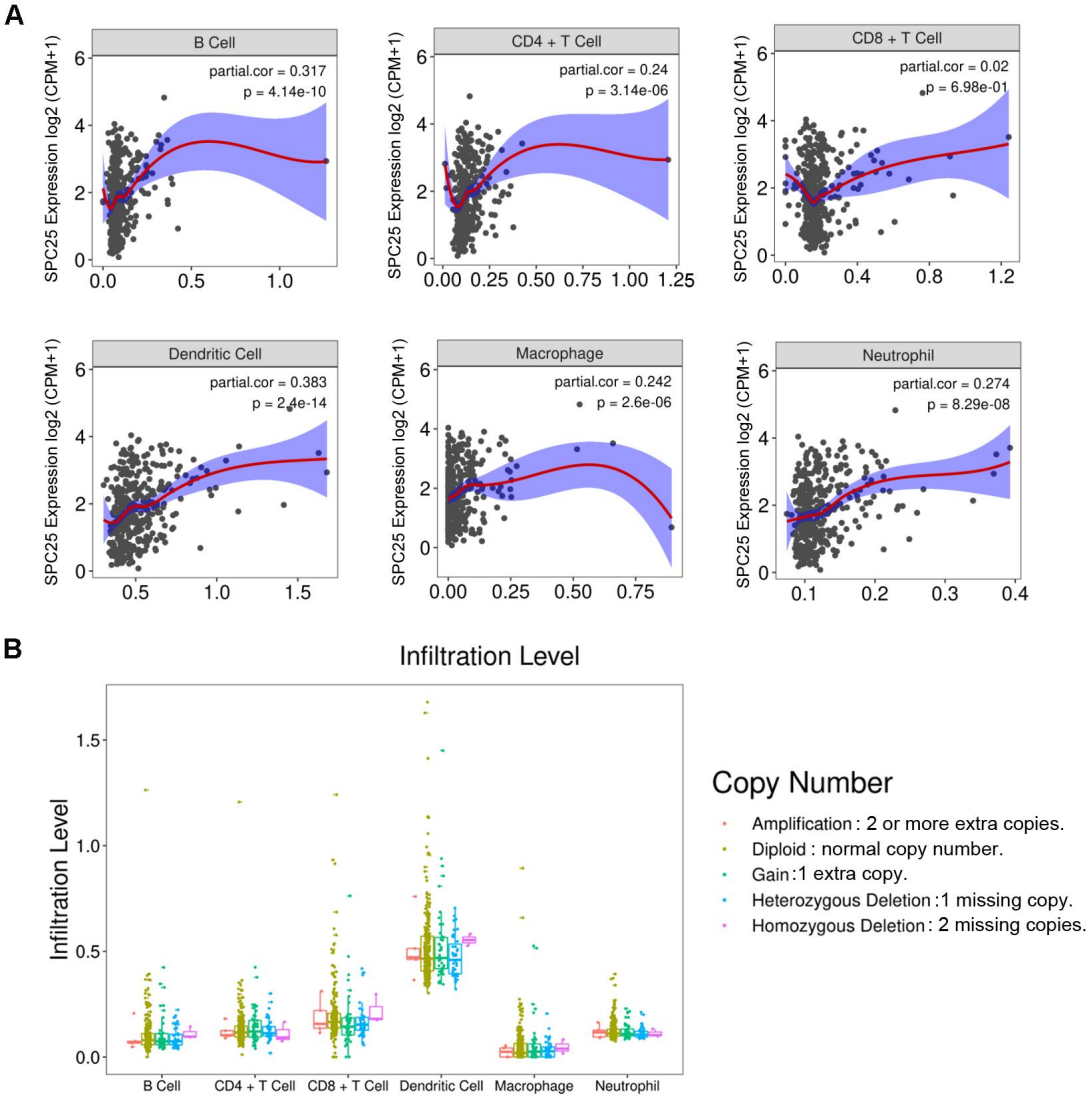
(**A**) Correlations between SPC25 expression and six types of immune cells infiltrated in HCC tissues. CPM: Counts of exon model per million mapped reads. (**B**) The infiltration level of six types of immune cells classified by the copy number variance (CNV) of the SPC25 gene.

Increasing evidence indicates that the infiltrating level of immune cells is close to tumor development and progression. Therefore, the relationship between SPC25 expression and immune cells infiltrated in the HCC tissues was studied. The result showed that SPC25 is significantly related to B cells, CD4^+^ T cell, dendritic cell, macrophage, and neutrophil (P <0.001, Figure 8A). In contrast, the number of CD8^+^ T cell is not associated with SPC25 (P > 0.05, Figure 8A). Figure 8B showed the copy number variance (CNV) classification of the SPC25 gene at the immune infiltration level of these six types of immune cells. All databases used for analyses in this article are listed in Table 2.

**Table 2:** Summary of databases used in this study.

**Name**	**link**	**This study**	**Key words**
TCGAportal	http://www.tcgaportal.org	To investigate the expression of SPC25 inhuman tissues	gene expression;28 cancer types; survival curve; DNA methylation; mutation
UALCAN	http://ualcan.path.uab.edu/	To analyze the SPC25 mRNA expression in different races, ages, molecular subtypes of HCC patients	gene expression; 16 cancer types; survival curve; DNA methylation
Human Protein Atlas	https://www.proteinatlas.org/	To detect SPC25’s distribution and HCC subcellular localization	proteins distribution; suHCCellular localization; impact for survival
Kaplan-Meier Plotter	http://kmplot.com/analysis/index.php?p=background	To analyzed the correlations between SPC25 mRNA expression and DMF, RFS as well as OS	breast cancer; subtype; survival curve;
Cistrome DB Toolkit	http://dbtoolkit.cistrome.org	Selected the TF of SPC25 – POLR2A	transcription factor; histone modifications
MEXPRESS	https://mexpress.be/	To unearth the methylation details of SPC25 as well as the relationship between SPC25 mRNA expression and different clinical characteristics of HCC	gene expression; 34 cancer types; DNA methylation
GEPIA 2	http://gepia2.cancer-pku.cn/#index	To assess the correlations between genes	gene expression; survival curve; isoform details; genes correlation; similar genes detection
LinkedOmics	http://www.linkedomics.org/admin.php	To predict the miRNAs related to SPC25	32 cancer types; mRNA / protein expression; target genes; enrichment analysis
TargetScanHuman	http://www.targetscan.org/vert_71/		gene; miRNA
starBase v3.0	https://bio.tools/starbase	To predict the miRNAs related to SPC25;	miRNA target; ceRNA network; RBP target; RBP motif; Pathway; Pan-cancer
STRING	https://string-db.org/cgi/input.pl	To obtain the interaction network between SPC25 and other important proteins	protein details; interactive network; functional enrichment
DISIDB	http://cis.hku.hk/TISIDB/index.php	To analyze the correlations between expressions of SPC25 mRNA and 3 kinds of immune factors	immune system; 30 cancer types; immunotherapy

## Discussion and Conclusions

In this study, we report that SPC25 is overexpressed in a variety of human cancers. We further characterize the expression of SPC25 mRNA and protein in TCGA and Chinese HCC samples. Survival analysis revealed that higher SPC25 mRNA and protein expression are associated with poor OS in HCC. To further explore the effects of overexpression of SPC25 on HCC tumor growth and progression, *in vitro* and *in vivo* function assays were performed. Our results demonstrate that SPC25 may promote HCC tumor growth by accelerating the cell cycle.

There is an uneven distribution of liver cancer burden throughout the world [28]. Due to regional and racial factors, more than 50% of liver cancer cases occur in China alone [3]. Therefore, in this study, we investigated the mRNA and protein expression of SPC25 in both TCGA samples and Chinese HCC specimens. Data from the TCGA showed that SPC25 mRNA levels are strikingly higher in Asian HCC patients than Caucasian patients. Furthermore, high SPC25 mRNA expression contributed more significantly to a greater risk of poor OS among Asians than Caucasians. While most HCC samples from the Human Protein Atlas displayed negative staining, elevated SPC25 protein levels were detected in 151/223 (67.7%) of Chinese HCC samples compared with adjacent non-tumor tissue.

Further survival analysis revealed that high SPC25 protein expression was significantly associated with shorter OS and PFS in Chinese HCC patients. Our study indicated that elevated SPC25 mRNA levels are observed in HCC patients globally, but enhanced protein expression is more likely to be observed among Chinese HCC cases. The factors that contribute to this divergent expression pattern based on region and race need further exploration.

The risk factors for HCC vary by geographical region [28]. In Asia, HBV infection is a significant risk factor [29]. As shown in Figure 2D, we observed a slightly higher hazard ratio for poor OS (2.94) in the high SPC25 vs. low SPC25 in patients with no HBV infection was observed, compared to patients infected with HBV (2.19). Interestingly, the hazard ratio was 8.97 in the high SPC25 group vs. low SPC25 group among Asians with no HBV infection, more than twice the hazard ratio for Asians infected with HBV (4.2). Previous studies pointed out that the molecular mechanism involved in non-HBV-related HCC is distinct from that of HBV-related HCC [30]. Further studies will elucidate the role of SPC25 in non-HBV-related HCC, especially among Asians.

We explored the DNA methylation status of SPC25 in HCC and found that SPC25 expression was strongly linked to DNMT expression. New methods, including epigenetic therapy using histone deacetylase inhibitors (HDACi), are currently being evaluated [31]. HDAC inhibitors have demonstrated antitumor efficacy by blocking cell cycle progression, inducing apoptosis, promoting differentiation, and reducing invasion and metastasis in tumor cells. Our data indicate that HCC tumorigenesis may be regulated by DNA modifications that alter the transcriptional levels of the SPC25 gene and support HDAC inhibitors' potential as promising treatments for HCC patients.

Weighted gene co-expression network and bioinformatic-based analyses revealed that SPC25 plays a role in the cell cycle, mitosis, and organelle organization, common pathways of tumor growth [32, 33]. SPC25 colocalized with kinetochores from prometaphase through anaphase, the knockdown of which resulted in mitotic arrest [34]. More specifically, SPC25 RNAi induced M phase arrest and cell death [34]. SPC25 RNAi also led to precocious polar body extrusion, resulting in severe chromosome misalignment and aberrant spindle formation [35]. qRT-PCR and western blot analysis showed that SPC25 silencing in HEPG2 cells significantly down-regulated cell-cycle associated proteins, including CyclinA2 and CyclinB1.

Accumulating evidence demonstrates that interactions between tumors and immune cells are imperative for tumor initiation and progression. Long, J et al. developed a TP53-associated immune prognostic model for HCC prognosis [36]. The type and quantity of tumor-infiltrating lymphocytes (TILs) that contribute to the immune environment should be assessed when choosing the best immunotherapy for HCC patients. SPC25 expression was found to be highest in Treg cells, which can promote tumor progression [37, 38]. Therefore, inhibitors targeting the immune microenvironment may contribute to a better therapeutic effect in HCC patients.

In conclusion, we found that SPC25 is significantly elevated in HCC tissue. Overexpression of SPC25 promoted HCC growth by accelerating cell-cycle progression. These results help elucidate molecular pathways of HCC carcinogenesis. Our study demonstrates that SPC25 may serve as a novel biomarker for HCC prognosis and as a potential target to treat HCC patients.

## MATERIALS AND METHODS

### Patients and tissues

One hundred five pairs of HCC tumor and nontumor samples were collected at Sun Yat-Sen University Cancer Center, China, from 2003 to 2009, for quantitative real-time polymerase chain reaction (qRT-PCR) analysis. Also obtained from Sun Yat-Sen University Cancer Center were formalin-fixed paraffin-embedded HCC tissue samples from 223 HCC patients. All patients were definitively diagnosed with HCC by two independent pathologists, and none of them had received preoperative treatments before surgery. In this study, no patients received preoperative therapies before surgery. Complete clinicopathologic data and follow-up information were gathered from the medical records of every patient.

### Ethics statement

Ethical approval was obtained from Sun Yat-Sen University Cancer Center's institutional review board before the project started. Each patient signed the written informed consent. All procedures were performed following the ethical guidelines of the Helsinki Declaration of 1975, which was revised in 2008.

### SPC25 mRNA expression analysis

GTEx (https://www.gtexportal.org/home/) and BioGPS (http://biogps.org/) [39] resources were used to investigate the expression of SPC25 in normal human tissues. SPC25 mRNA levels in cancer were summarized using UALCAN (http://ualcan.path.uab.edu/) [40]. UALCAN was also applied to compare SPC25 expression based on gender and race. The Wilcoxon rank-sum test was used to assess the significance of observed differences.

### Quantitative real-time reverse transcription-polymerase chain reaction (qRT-PCR)

Total RNA was extracted from tissues or cell lines using TRIzol reagent (Invitrogen). RNA was reverse-transcribed using an Advantage RT-for-PCR Kit (Clontech Laboratories) according to the manufacturer's instructions. The β-actin gene was used as an internal control for qRT-PCR. qRT-PCR was performed using SYBR Green PCR Kit (Applied Biosystems) and Light-Cycler480 384-well PCR system (Roche Diagnostics). The conditions for qRT-PCR amplification were as follows: 42° C for 5 min, 95° C for 10 s, followed by 35 cycles of 95° C for 5 s, 60° C for 20 s, and 72° C for 15 s. Melt curve analysis was applied to determine the reaction specificity. Relative gene expression was calculated using the 2^−ΔΔCt^ method. Primers for SPC25 were 5’-TACGGACACCTCCTGTCAGA-3’ (sense) and 5’-GGGCACTATCTGACACTTCAT-3’(anti-sense). Primers for β-actin were 5’- TGGCACCCAGCACAATGAA-3’ (sense) and 5’- CTAAGTCATAGTCCGCCTAGAAGCA-3’ (anti-sense). The student's t-test was used to compare mean gene expression between two groups.

### Immunohistochemical (IHC) staining

Paraffin-embedded, formalin-fixed tissue slides were dewaxed by xylene, rehydrated with graded ethanol, rinsed with deionized water, and then blocked with 3% hydrogen peroxide for 10 min at room temperature. Antigen retrieval was carried out by high-pressure-cooking in 10 mM citrate buffer (pH 6.0) for 10 min. Slides were then blocked with 5% normal goat serum for 30 min at room temperature. Anti-SPC25 antibody (1:100, Proteintech, China, Catalog: 26474-1-AP) was subsequently incubated with tissue sections at 4° C overnight. Slides were then incubated with an Envision detection system (DAKO), and Meyer's hematoxylin counterstained the nucleus.

### Survival analysis of SPC25 mRNA in HCC

UALCAN (http://ualcan.path.uab.edu/) was used to determine the relationship between SPC25 expression and overall survival in HCC. Subgroup survival analysis of gender and race was performed using UALCAN. Kaplan-Meier Plotter (http://kmplot.com/analysis/index.php?p=background) was used to examine correlations between SPC25 expression and overall survival (OS) of HCC patients based on the different subgroups. Hazard ratios with 95% confidence intervals and log-rank p-values were calculated.

### Cell lines and culture conditions

Two HCC cell lines (PLC8024 and HepG2) were obtained from the Institute of Virology, Chinese Academy of Medical Sciences (Beijing, China). Huh7 was purchased from American type culture collection (ATCC, Manassas, VA, USA). Cells were cultured in Dulbecco's modified Eagle medium (DMEM; Gibco BRL, Grand Island, NY, USA) supplemented with 10% fetal bovine serum (FBS; Gibco BRL, Grand Island, NY, USA). All cells were cultured at 37° C in a humidified incubator containing 5% CO_2_.

### Western blot analysis

Equal amounts of protein were separated by 12% dodecyl sulfate, sodium salt (SDS)-Polyacrylamide gel electrophoresis (PAGE) and transferred to microporous polyvinylidene difluoride (PVDF) membranes (Roche, UK). Membranes were incubated with antibodies against SPC25 (1:1000, Proteintech, China, Catalog: 26474-1-AP), β-actin (1:2000, CST, USA, Catalog: #3700), CDK1 (1:1000, CST, USA, Catalog: #9116), cdc25A (1:1000, CST, USA, Catalog: #3652), Cyclin A2 (1:1000, CST, USA, Catalog: #4656), Cyclin B1 (1:1000, CST, USA, Catalog: # 12231), CyclinD1 (1:1000, CST, USA, Catalog: # 55506), and CyclinE1 (1:1000, CST, USA, Catalog: #4129). Membranes were then incubated with secondary antibodies (1:1000) conjugated to horseradish peroxidase (HRP) at room temperature for 60 min. Finally, blots were visualized using a Luminata^TM^ Crescendo Western HRP Substrate (Millipore, USA). β-actin was used as a loading control.

### Functional assays *in vitro*

Cell proliferation assays, foci formation assays, and soft agar formation assays were used to explore the biological function of SPC25 in HCC *in vitro*. According to the manufacturer's protocol, cell proliferation assays were carried out using the Cell Counting Kit-8 (Dojindo, Kumamoto, Japan). For foci formation assay, 1×10^3^ cells were seeded in 6-well plates and cultured at 37° C for one week to allow colony formation. Dishes were washed with PBS, fixed with 4% (paraformaldehyde), and stained with 0.1% crystal violet. The number of colonies (>50 cells/colony) was counted. Soft agar colony formation assays were performed to evaluate the role of SPC25 in non-adherent colony formation. Briefly, 5 ×10^3^ cells were kept in suspension in a soft agar mixture, consisting of DMEM, 10% fetal bovine serum, and 0.35% Sea Plaque agarose. The suspension mixture was overlaid on a solidified 0.5% agar base. Colonies were captured and counted by a fluorescence microscope at ten fields of view per well. All results were expressed as the mean ± SD of triplicate independent experiments.

### Functional assays *in vivo*

4-week old male BALB/C nude mice were obtained from Guangdong Animal Center (Guangzhou, China) and kept under specific pathogen-free conditions. Each nude mouse was injected subcutaneously in the right flank with SPC25 overexpressing cells (2 × 10 ^6^ cells for Huh7-SPC25; 2 × 10^6^ cells for PLC8402-SPC25), and control cells (Huh7-Vec, PLC8402-Vec) were subcutaneously injected into the left dorsal flank, respectively. Tumor size was measured and calculated as tumor volume (mm^3^) = 1/2 (a×b^2^). About three weeks later, the tumors were excised, weighed, and fixed in a formaldehyde solution. All animal experiments were conducted following the Institutional Animal Care and Use Committee guidelines at Sun Yat-sen University Cancer Center.

### Spc25 siRNA transfections

Two small interfering RNA oligonucleotides against SPC25 and a non-targeting control siRNA (si-NC) were purchased from GeneCopoeia Company (Guangzhou, China). All siRNAs were transfected into the HepG2 cell line using Lipofectamine 2000 reagent (Invitrogen, USA) and Opti-MEM (Gibco, USA). Cells were harvested for evaluation 48 h after transfection. The target sequences of the si-SPC25 oligonucleotides were as follows: si#1: 5′- GGUGAGAAAUUGCAGUUUAUU -3′ and si#2: 5-AAGCGAAUGCAGAGAGGUUGA-3.

### TF identification

The Cistrome DB (http://dbtoolkit.cistrome.org) is a comprehensive resource for searching transcription factors (TFs) that bind to cis-regulatory elements of genes of interest [41]. The Cistrome DB Toolkit was used to predict TFs that are likely to increase SPC25 expression in HCC.

### DNA methylation modification analysis

MEXPRESS (https://mexpress.be/), a data visualization tool, allows users to investigate the relationships between multiple factors, including TCGA gene expression, DNA methylation status, and clinical and pathological parameters [28]. MEXPRESS was used to determine the methylation status of the SPC25 gene.

### Identification of miRNAs that target SPC25

TargetScanHuman (http://www.targetscan.org/vert_71/) can predict miRNAs that may target specific genes. starBase v3.0 (https://bio.tools/starbase) allows for the multiple interactions of miRNA-mRNA, RNA-RNA, miRNA-ncRNA, ncRNA-RNA, RBPncRNA, and RBP-mRNA inferred from CLIP-seq, degradome-seq, and RNA-RNA interactome data. These three databases were used to identify miRNAs that are likely to regulate SPC25 mRNA. LinkedOmics (http://www.linkedomics.org/admin.php) is an open-access platform for analyzing multi-omics bioinformatic data within and across 32 cancer types. These resources were used to explore the relationship between SPC25 and miR-451 expression.

### Dual-luciferase reporter gene assay

The putative binding sites between miR-451a and SPC25 were identified with the bioinformatics website (micro-RNA.org). The pMIR-REPORT plasmids (Ambion, Carlsbad, CA, USA), SPC25 3′ untranslated region (UTR), and 3′UTR mutant fragments were inserted through XbaI and SacI. The wild-type or mutant reporter constructs were co-transfected into the plated Escherichia coli DH5α cells (Takara, Dalian, Liaoning, China). When the cell reached 90–95% confluence, SPC25 3′UTR pMIR-REPORT, 3′UTR mutant-pMIR-REPORT, or miR-451a mimic (GenePharma Ltd., Shanghai, China) or negative control mimic were transfected using Lipofectamine 2000 transfection (Invitrogen Inc., Carlsbad, CA, USA). The light intensity was determined based on the protocols of the Dual-Luciferase® Reporter Assay System (Promega). The assay was independently repeated three times.

### Gene correlations analysis

GEPIA2 (http://gepia2.cancer-pku.cn/#index) is an open resource to query gene classes and isoform classes exhibited in different cancer subtypes from the TCGA and GTEx projects among 60, 498 genes and 198, 619 isoforms. Multiple analyses can be performed, including differential expression analysis in tumor/normal, expression profiling based on cancer type or pathological stage, survival analysis, and correlation analysis. GEPIA2 was used to assess correlations between genes of interest in HCC.

### Protein-protein interaction and functional enrichment analysis

STRING (https://string-db.org/cgi/input.pl) is a database that predicts protein-protein interactions via direct (physical) or indirect (functional) ways [30]. The interaction map between SPC25 and other proteins was derived using STRING.

### Immune-related analysis

DISIDB (http://cis.hku.hk/TISIDB/index.php) is a free web tool that contains multiple heterogeneous data types to analyze cancer and immune system interactions [31]. Here, DISIDB was used to analyze Spearman correlations between SPC25 expression, immune cells, MHCs, immune inhibitors, and immune stimulators.

### Statistical analysis

All statistical analyzes were performed using the Statistical Package for the Social Sciences (SPSS) software version 16.0 (SPSS Inc, Chicago, IL). Paired two-tailed student's t-test was used to compare the expression of SPC25 in primary HCC tumors and corresponding adjacent non-tumor tissue. The chi-square test or Fisher's exact test was used to assess the correlation between SPC25 expression and clinicopathological parameters. Disease-specific survival was calculated from surgery to either the time of death from HCC or the last follow up (31 December 2014). The predictive value was calculated by Kaplan-Meier analysis with the log-rank test. Univariate and multivariate survival analysis was performed using the Cox proportional hazard model with a stepwise forward procedure (the entry and removal probabilities were 0.05 and 0.10, respectively). Differences were considered statistically at a P-value of P < 0.05.
